# Exciton Diffusion to Low Energy Sites of the Acceptor Drives Charge Photogeneration in D18:Y6 Solar Cells

**DOI:** 10.1021/acs.jpcc.4c06706

**Published:** 2024-10-31

**Authors:** Thomas Sayner, Arvydas Ruseckas, Jonathon R. Harwell, Ifor D. W. Samuel

**Affiliations:** Organic Semiconductor Centre, SUPA, School of Physics and Astronomy, University of St. Andrews, North Haugh, St. Andrews, Fife KY16 9SS, U.K.

## Abstract

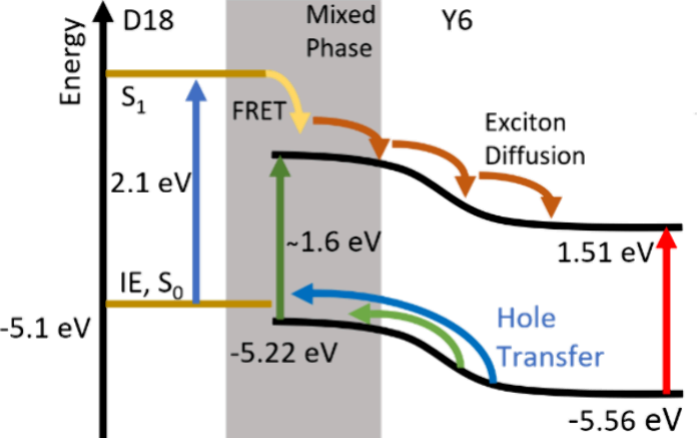

We have investigated charge generation pathways in efficient organic photovoltaic blends of the polymer donor D18 and the small-molecule acceptor Y6 using transient absorption and time-resolved fluorescence spectroscopies. We find that energy transfer from D18 to Y6 outcompetes electron transfer and is followed by exciton diffusion from regions of the disordered Y6 phase to Y6 aggregates before hole transfer to D18. Aggregation of Y6 molecules increases their ionization energy by ∼0.3 eV and provides a driving force for hole transfer from Y6 excitons and spontaneously generated charge pairs to D18. We observed ultrafast depolarization of the Y6 ground-state bleaching in <200 fs, which indicates delocalization of primary excitons in Y6 aggregates. This delocalization can explain the spontaneous generation of charge pairs in neat Y6 films and Y6-rich blends. Our results show that subtle aggregation control of the low-energy absorber can be used for balancing photocurrent generation with low voltage loss in photovoltaic blends.

## Introduction

Organic photovoltaic (OPV) devices can be made into thin and flexible solar panels using high-throughput roll-to-roll coating and have seen rapid increases in efficiency over the past decade largely due to the development of nonfullerene electron acceptors (NFAs).^1,2^ Current efforts are mainly focused on improving long-term operational stability by developing photostable materials and morphologies.^3−6^ In order to develop more stable materials, it is important to understand the processes underlying efficient performance. Only a few OPV materials show a power conversion efficiency (PCE) above 18% under AM1.5 conditions. Lab-scale single-junction cells with a record-high PCE of 18.2% (institution-certified 17.6%) have been reported in 2020 using blends of the electron donor D18 and the electron acceptor Y6 prepared from solution.^7^ Recently, solar cells with PCE around 19% have been reported using ternary blends of D18 with other high-performing polymer donors PM6 and a Y-series electron acceptor as well as by using additives to tune the morphology and performance of D18 blends with other Y-series electron acceptors.^8,9^

A unique feature of photovoltaic blends with Y-series acceptors is barrierless free carrier generation despite a low energy offset at the heterojunction.^10^ It has been suggested that the electrostatic potential formed at the heterojunction by the molecular quadrupole moments of the aligned acceptor molecules can effectively compensate for the Coulomb attraction in the interfacial charge transfer (CT) state and lead to its dissociation into free carriers.^11,12^ Solar cells with Y-series acceptors show substantially reduced nonradiative voltage loss as compared to other acceptors, which can be explained by the hybridization between the interfacial CT state and local exciton states that enhances the radiative recombination rate.^13−15^

There are several possible pathways for free carrier generation in OPVs with NFAs that allow some flexibility for material design and morphology refinement. Following excitation of the donor, electron and energy transfer to the acceptor can both be very fast and competing processes.^16−19^ When the acceptor is excited, hole transfer to the donor is usually slower because of the lower driving force and larger acceptor domains so that excitons have to diffuse to reach the heterojunction.^20−22^ Spontaneous exciton splitting into electron–hole pairs has been reported in neat Y6 films, which can play an important role in photocurrent generation, particularly in semitransparent cells with a low donor content.^23−26^ Exploring charge generation mechanisms and efficiency in relation to blend morphology and energy offsets improves the understanding of device physics and helps in further development.

Here, we show that the formation of low-energy Y6 aggregates provides a driving force for hole transfer, which is crucial for charge generation in D18:Y6 blends. Ultrafast depolarization measurements indicate exciton delocalization in Y6 aggregates, which can help in charge generation in Y6 domains.

## Experimental Methods

### Film and Device Preparation

D18 and Y6 were supplied by 1-Material. The perylene diimide derivative PDINN was purchased from Ossila, and poly(9-vinylcarbazole) (PVK) was supplied by Sigma-Aldrich. Blends were fabricated by dissolving D18/PVK and Y6 together in chloroform at 11 mg/mL in various ratios, stirring at 50 °C for 1 h, and then spin-coating onto a glass substrate at 1500 rpm, resulting in an approximately 130 nm thick film. Neat Y6 was made similarly from a 15 mg/mL solution. Blend films were solvent vapor-annealed in a closed container with chloroform vapor for 5 min and then encapsulated, all in the nitrogen environment of the glovebox. Devices were made with a structure of ITO/PEDOT:PSS/D18:Y6/PDINN/Ag. PEDOT solution was spin-coated at 4000 rpm for 30 s onto the ITO substrate and then annealed at 150 °C. A layer of D18:Y6 solution was then deposited by spin-coating at either 4000 or 1500 rpm, with half of the samples solvent vapor annealed in chloroform for 5 min afterward. This resulted in active layers of around 40 nm (4000 rpm) and 130 nm (1500 rpm). A layer of 1.5 mg/mL PDINN solution was then deposited at 1500 rpm, followed by evaporating a top silver electrode. The *J*–*V* curves of the cells were measured under AM1.5G illumination (100 mW/cm^2^) with a pixel area of 0.05 cm^2^. Film thicknesses were measured by using spectroscopic ellipsometry.

### Steady-State and Transient Absorption

Ground-state absorption spectra were measured using a DS5 spectrophotometer from Edinburgh Instruments. Transient absorption (TA) spectra were measured with a HARPIA spectrometer from Light Conversion. For excitation, we used 180 fs pulses from a PHAROS regenerative amplifier in combination with an ORPHEUS OPA at a 50 kHz repetition rate, both from Light Conversion, while the white light probe was generated using 1030 nm light in a sapphire plate. Transient anisotropy dynamics were measured with a one-color pump–probe technique using the output from the ORPHEUS OPA and silicon photodiodes as detectors.

### Time-Resolved PL, PLQY, Spectroscopic Ellipsometry, EQE, and Ionization Energies

Time-resolved photoluminescence was measured using a Hamamatsu C10910-05 streak camera in the synchroscan mode following excitation by the second harmonic of a PHAROS oscillator operating at a pulse repetition rate of 80 MHz with a very low excitation density of ∼10^15^ cm^–3^. The amplified pulses at a 100 kHz repetition rate were used to measure PL at higher excitation densities for comparison with TA. Ionization energies (IEs) were measured using a KP Technology APS03 Kelvin probe system measuring in the ambient-pressure photoemission spectroscopy (APS) mode.^27^ PLQY was measured using a Hamamatsu C9920-02 integrating sphere in a flowing N_2_ atmosphere and excitation wavelengths of 515 nm (D18) and 840 nm (Y6). Spectroscopic ellipsometry was performed using a J. A. Woollam M-2000 ellipsometer. All measurements were conducted at room temperature. The external quantum efficiency (EQE) measurements were performed at zero bias by illuminating the device with monochromatic light supplied by a xenon arc lamp in combination with a Bentham TMc300 monochromator. The number of photons incident on the sample was calculated for each wavelength by using a silicon photodiode calibrated by the National Physical Laboratory (NPL).

## Results

### Steady-State Absorption and Devices

**Figure 1:**
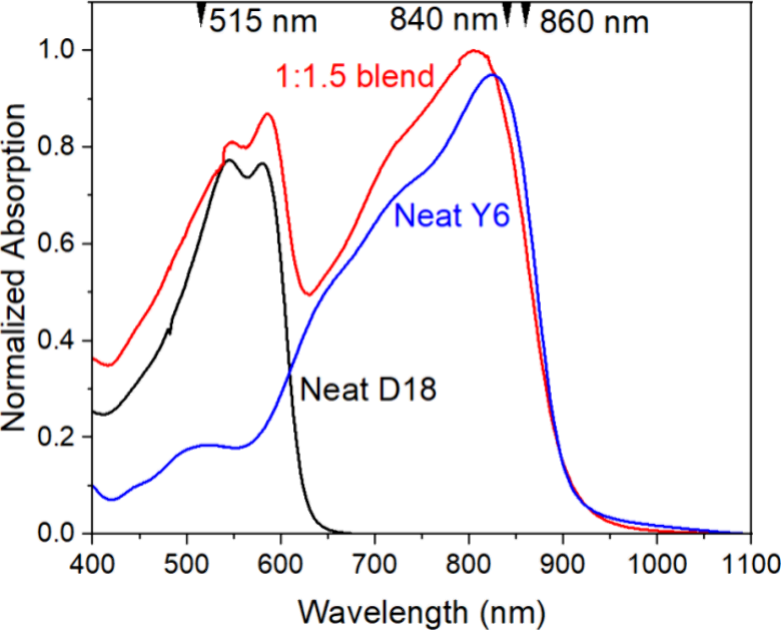
Ground-state absorption in films of 1:1.5 D18:Y6, neat Y6, and neat D18. Also indicated are the excitation wavelengths used to study these materials.

Figure 1 shows the ground-state absorption spectrum of a 1:1.5 D18:Y6 blend compared with neat D18 and Y6 films. D18 absorption dominates in the region of 400–600 nm, while the Y6 absorption maximum is at 830 nm in the neat film and at 810 nm in the blend. We studied charge generation using different excitation wavelengths, as indicated. We also made solar cells using the blend prepared under the same conditions. The best cells showed a PCE of 15.9% under AM1.5 conditions (Table S1 and Figure S1 in the SI). This is just slightly lower than the best-reported certified efficiency of 17.6%.^7,28^ Absorption spectra of blends with different D18:Y6 ratios are shown in Figure S2.

### TA Spectra

**Figure 2:**
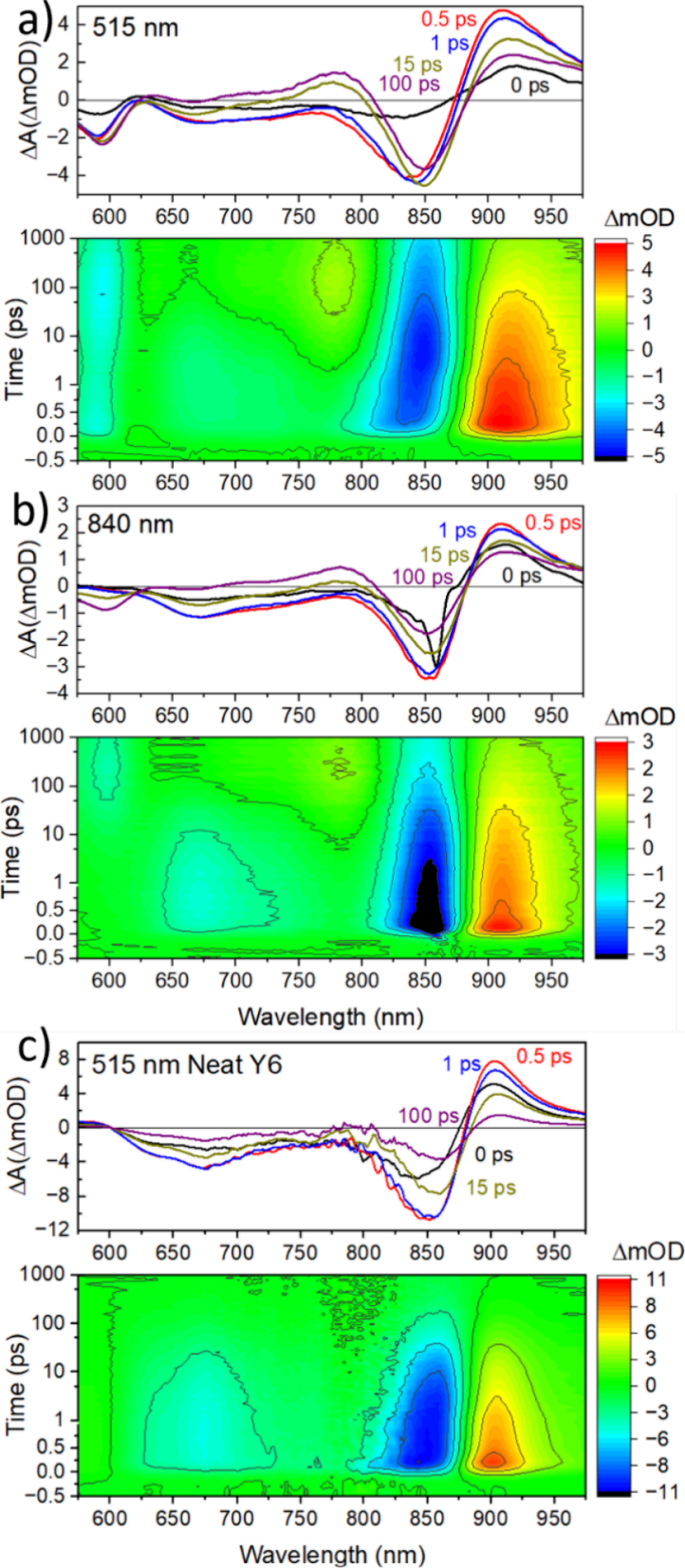
TA spectra for the D18:Y6 1:1.5 blend with excitation at (a) 515 nm with a density of 4.8 μJ/cm^2^, (b) at 840 nm with 2 μJ/cm^2^, and (c) neat Y6 at 515 nm excitation, 12 μJ/cm^2^.

TA spectra for the 1:1.5 D18:Y6 blend measured with two different excitation wavelengths are shown in Figure 2a,b. Excitation light of 515 nm is absorbed predominantly by D18, while 840 nm light falls on the low-energy side of Y6. In both cases, the TA spectra show two main bands of decreased absorption: one at 570–620 nm, which is dominated by ground-state bleaching (GSB) of D18, and the other at 770–870 nm, which represents a GSB of Y6 (cf. the TA spectra of the neat films in Figures 2c and S3a,b). In the case of 515 nm excitation of the blend, D18 GSB decreases in ∼2 ps and then increases again to reach its maximum at about 100 ps. The Y6 GSB peak shows a dynamic red shift in about 2 ps. In the case of 840 nm excitation, the GSB of D18 grows slower than that with 515 nm excitation.

### Analysis

**Figure 3:**
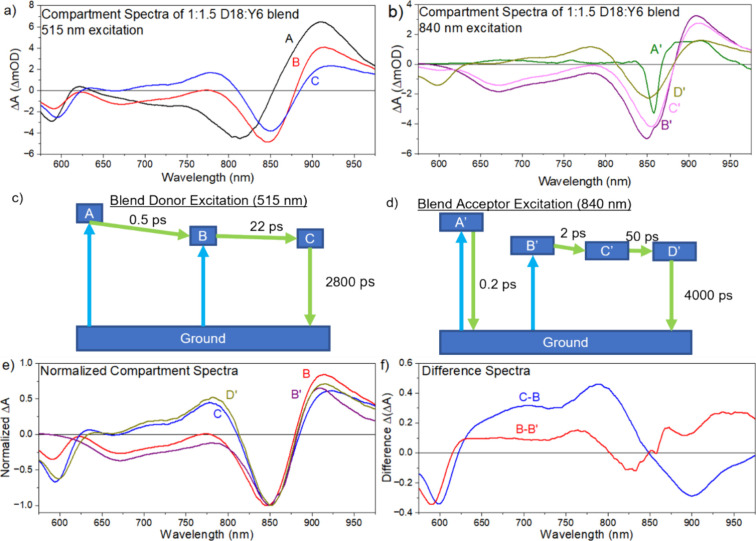
EAS and time constants obtained from global analysis of the TA spectra in the 1:1.5 blend for 515 nm excitation (a, c) and for 840 nm excitation (b, d). Selected EAS normalized at around 830 nm (e) and the difference spectra between normalized EAS (f).

We globally analyzed the TA spectra using a kinetic model consisting of sequentially interconverting evolution-associated spectra (EAS), i.e., **A** → **B** → **C** → ··· Here, the first arrow represents exponential decay of **A** and growth of **B** with the same time constant and so on. We used the minimum number of EAS needed to reproduce the main features of the TA spectra (Figure 3).

For 515 nm excitation, we used a three-compartment model with fixed excitation of the first two compartments **A** and **B** in a 3:1 ratio. This was determined by the ratio of the ground-state absorption of D18 and Y6 at 515 nm. Spectrum **A** shows the GSB of D18 at ∼590 nm and the GSB of Y6 with a maximum at ∼820 nm (Figure 3a). The intensity of Y6 GSB is higher than D18 despite the lower fraction of excitation light absorbed by Y6 (0.25). This indicates that energy or electron transfer occurs from D18 to Y6 within the 200 fs response function of our setup. Spectrum **A** evolves in 0.5 ps into spectrum **B,** showing a decrease of D18 GSB as compared to **A**, as well as an increase and a red shift of Y6 GSB. The drop in the intensity of the D18 GSB on this time scale suggests the presence of Förster resonance energy transfer (FRET) from D18 to Y6 rather than electron transfer. Some electron transfer may still occur in competition with FRET and photoinduced absorption (PIA) by charges may cause an apparent decay of the D18 GSB. Further analysis showed that FRET dominates over electron transfer (vide infra). During the subsequent 20 ps (**B → C** evolution), the D18 GSB increases again, indicating hole transfer from Y6 to D18, which is consistent with the photoluminescence quenching of Y6 on this time scale (Figure S4b). A broad PIA band appears at 670–810 nm, which can be attributed to photogenerated charges, mainly dominated by absorption of the Y6 anion. We also observe that the induced absorption around 900–970 nm is much longer-lived than in the neat films. This may be due to the contribution from the D18 cation, which was shown to absorb in this region in blends with PC_60_BM by Wang et al.^29^ The TA spectra of the neat films can be reproduced with two EAS of each. They show a slight change in the excited-state absorption of D18 and a red shift of Y6 GSB over time (Figure S5). The χ^2^ goodness of fit of the TA global analysis is shown in Table S2.

The TA spectra for the 840 nm excitation were reproduced using four EAS (Figure 3b,d) with fixed excitation of the first two compartments **A′** and **B′**. Compartment **A′** shows a narrow negative peak, which is only present during the temporal overlap of the pump and probe pulses; therefore, it is attributed to a coherent artifact.^30^ It is caused by diffraction of the pump from a photoinduced transient grating generated by the interference of the pump and probe pulses. The diffracted pump propagates in the same direction as the probe light and enhances the measured probe signal. **B′** meanwhile reproduces the initial Y6 GSB and converts to **C′** in 2 ps, showing the formation of a small D18 GSB. Over the next 50 ps (**C′** → **D′** evolution), the signal of the D18 GSB increases further, indicating hole transfer from Y6 to D18. Normalized **C** and **D′** spectra are nearly identical (Figure 3e), suggesting that the same types of excited species are formed after 50 ps following 515 and 840 nm excitation. In this case, they represent holes in the D18 domain and electrons in the Y6 domain. Their lifetime extends beyond the time range of our TA measurements, which is limited to 1 ns.

No significant D18 GSB is observed in spectrum **B′,** indicating that hole transfer to D18 occurs at later times with 840 nm excitation. In this case, **B′** represents mainly Y6 excitations in the blend and serves as a convenient baseline for assessing other excited species formed by the 515 nm pump. The difference **B–B′** calculated after normalizing these spectra at 850 nm can be considered to represent these other species because the contribution of Y6 GSB is subtracted. Similarly, the difference spectrum **C–B** at 620–850 nm can be attributed to charge absorption generated by hole transfer assuming that the change in the Y6 GSB spectrum upon hole transfer is not significant. The negative **C–B** difference at 850–970 nm is the result of the lower absorption by photogenerated charges as compared to that of Y6 excitons. We notice that the shape of **B–B′** is very different from **C–B** but resembles the TA spectra of neat D18 films. This suggests that **B–B′** is dominated by D18 excitons (Figure S5). The dip in **B–B′** around 830 nm can be explained by a small difference in the shape of the Y6 GSB spectra with 515 and 840 nm excitation. To estimate an upper bound of the amount of charge generated by electron transfer from D18 to Y6, we compared the area of PIA at 600–800 nm in both spectra. The area for **C–B** is 3.6 times bigger than that for **B–B′**, indicating that at least 80% of the charges are generated by hole transfer after FRET from D18 to Y6, even when assuming a negligible contribution from D18 excitons to **B**. The FRET efficiency is higher than in blends of a polymer donor PM6 with different acceptors, where it was estimated to range between 30 and 66%.^19^ We carried out time-resolved PL and TA measurements also in the 1:2 D18:Y6 blend, which gave very similar results to the 1:1.5 blend, indicating that FRET dominates in the 1:2 blend too (shown in Figures S3c and S4). The dominant contribution of FRET over electron transfer in other blends has been inferred on the basis of the device efficiency dependence on the IE offset.^16,17^

The dominance of FRET over electron transfer is not surprising considering the strong tendency of D18 and Y6 to form crystallites as reported by Wang et al. using GIWAXS measurements.^28^ They observed strong diffraction peaks corresponding to π–π spacings of 3.74 Å for D18 and 3.57 Å for Y6 in the blend with coherence lengths along the π–π stack of 36 Å for D18 and 27 Å for Y6. The Y6 peaks were much stronger in the out-of-plane direction, indicating a preferred face-on orientation of Y6 molecules. They also observed strong extension of D18 chains in the plane of the film, showing both face-on and edge-on molecular orientations.

We estimated the average size of the D18 domains using the observed FRET rate. To account for the size of Y6 domains, we used a FRET model from a point dipole to a slab (details are in Supplementary note 1 in the SI). We calculated a critical transfer distance of 8.5 nm for FRET to the 2.7 nm thick Y6 slab from the spectral overlap and the PL quantum yield of D18. The slow FRET component of 1 ps gives us an average width of the D18 domain of ∼3.6 nm, which is identical to the π–π stacking coherence length of D18 chains found in the D18:Y6 blend by Wang et al. using GIWAXS measurements.^28^ This agreement indicates a similar blend morphology of our blends and the published work. The domain width of 3.6 nm corresponds to about nine stacked D18 chains, indicating phase separation on an intermediate scale in this blend.

### Hole Transfer to D18

**Figure 4:**
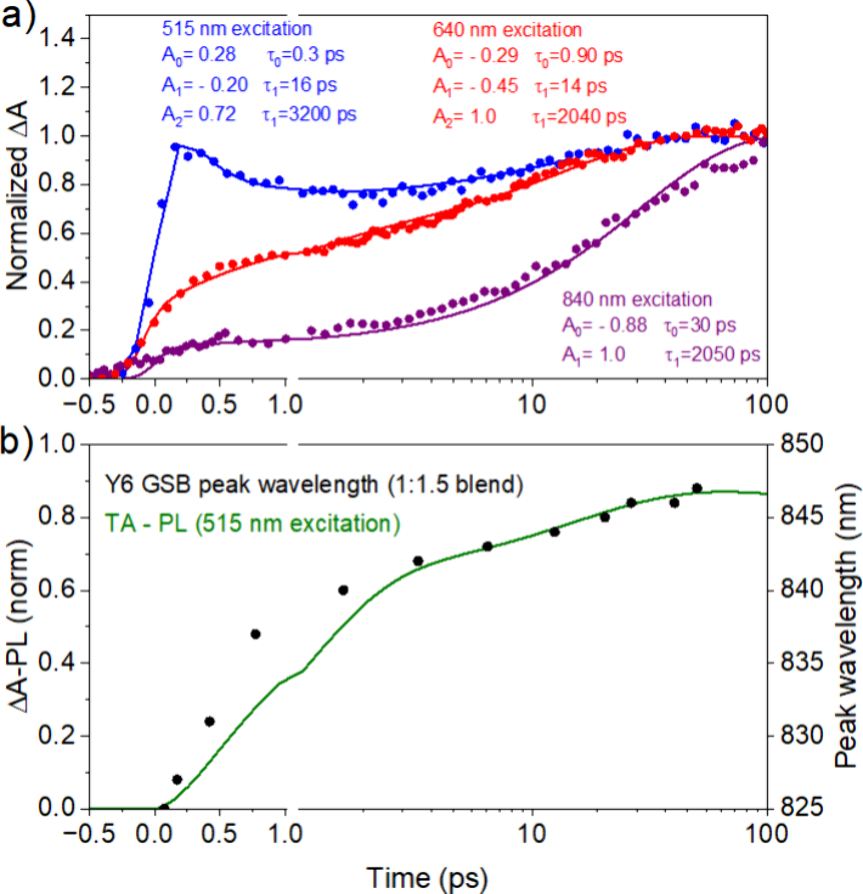
(a) TA kinetics of the D18 GSB at 590 nm in a 1:1.5 D18:Y6 blend for different excitation wavelengths. Solid lines represent the fits with a sum of exponential decay functions with the given amplitudes and time constants. (b) Kinetics of the D18 hole population after 515 nm excitation (TA-PL), and the peak shift of Y6 GSB (circles).

Since the neat Y6 film shows a very small TA signal at 590 nm (Figure S5), the signal in the blend at 590 nm is dominated by the GSB of D18 and can be used to track the population of photogenerated holes in D18 with different excitation wavelengths (Figure 4a). Excitation at 840 nm gives a slow rise of the D18 GSB with a time constant of ∼30 ps, which can be explained by exciton diffusion-mediated hole transfer from large Y6 domains. A faster rise of the D18 GSB occurs after 640 nm excitation (Figure S3d), including a component with a relative amplitude of 0.26 rising within our instrument response of ∼200 fs. This can be attributed to hole transfer from nonrelaxed or vibrationally hot Y6 excitons. It is followed by a slower rise, which can be fitted with the time constants of ∼1 ps and ∼14 ps to give an average hole transfer time of ∼9 ps. This is slightly slower than the hole transfer found in the PM6:Y6 blend (∼6 ps).^20,21^

The D18 GSB immediately after 515 nm excitation includes exciton and hole contributions. We used the deconvoluted PL decay of D18 recorded in the 1:1.5 blend as a measure of D18 exciton population and subtracted it from the D18 GSB at 590 nm (Figure S6a). The result of TA-PL is proportional to the hole population in D18 after 515 nm excitation and shows hole transfer dynamics similar to that with 640 nm excitation. It also closely follows the red shift of the Y6 GSB peak in this blend (Figure 4b). A very similar dynamic GSB red shift is seen in a blend with PVK and in neat Y6, but the peak occurs at a longer wavelength by ∼16 nm (Figures S3b,e and S6b). This indicates that the origin of the Y6 GSB red shift is exciton diffusion to lower energy sites and is not caused by the formation of the charge absorption band at around 780 nm.

The kinetics of the Y6 GSB in the 1:1.5 blend with 515 nm excitation (Figure S7) show a rise over the first 3 ps, which is a combination of energy transfer from D18 and exciton diffusion. This then decreases slightly over the next 100 ps, which is the time scale of hole transfer to D18. The decrease can be explained by the growing absorption of the Y6 anion and the D18 cation, which overlap with Y6 GSB as shown by Wang et al.^29^ We see very similar kinetics for the 1:2 blend at this excitation wavelength. The TA signals with direct excitation of Y6 at 640 and 840 nm show an instantaneous rise and a partial decay in ∼100 ps, which can be explained by the exciton–exciton annihilation and geminate recombination of spontaneously generated charge pairs in Y6 domains (vide infra)*.* The decay at >300 ps is due to charge recombination. We investigated exciton–exciton annihilation in neat Y6 at different excitation densities (Figure S8a). At 1.3 μJ/cm^2^, we observed no annihilation, whereas at 4.6 μJ/cm^2^, we saw that ∼27% of the excitons annihilate in 250 ps. Similarly, ∼ 54% is lost to annihilation at 12 μJ/cm^2^.

### Excitation Dynamics in Y6 Domains

**Figure 5:**
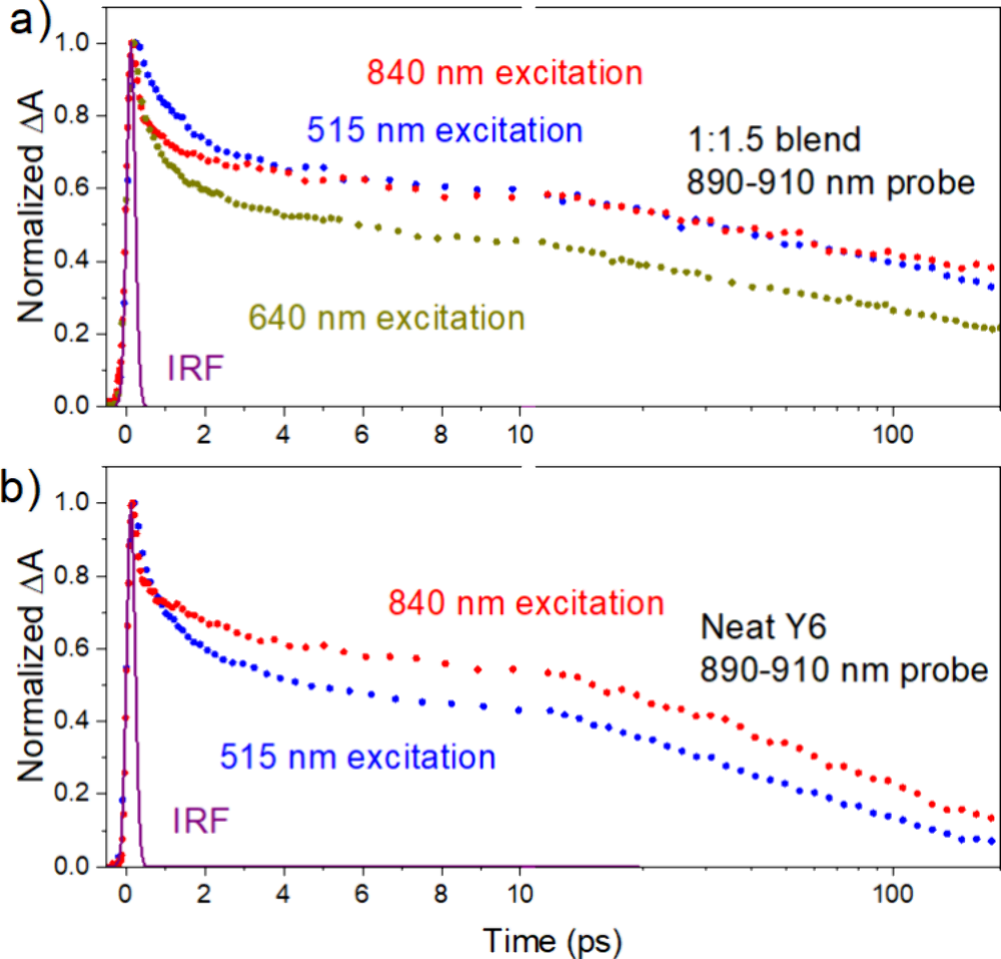
TA kinetics at 890–910 nm in a (a) 1:1.5 D18:Y6 blend and (b) neat Y6 film for different excitation wavelengths. The signal is dominated by Y6 excitons at an early time (<10 ps).

Previous reports indicated that a significant fraction of Y6 excitons can split into electron–hole pairs in neat Y6 films.^21,24,25^ We observe a small decrease of the Y6 GSB at 770–850 nm in neat films, which appears in 2 ps after excitation (Figure S5). The position of this dip coincides with the charge absorption band seen in the 1:1.5 blend (Figure 3f), suggesting that it may represent charge absorption overlapping with Y6 GSB. We observe partial decays of PIA at 890–910 nm in the 1:1.5 blend as well as the neat Y6 film in about 2 ps (Figure 5). There is a corresponding rise of the D18 hole population in the blend with 515 and 640 nm excitations (Figure 4), indicating that hole transfer to D18 occurs simultaneously with the decay of this PIA. It also closely follows the Y6 PL decay when the long-lived PIA component representing absorption by photogenerated charges is subtracted (Figure S8). Based on these observations, we can assign this PIA at an early times to Y6 excitons. It shows a fast partial decay after 840 nm excitation in ∼2 ps, which has no corresponding rise in the D18 hole population in the blend (Figure 5a). This indicates that some of the Y6 excitons decay without hole transfer to D18. The neat Y6 film also shows a fast PIA decay component similar to that of the blend (Figure 5b). These observations imply that some Y6 excitons split into charge pairs in Y6 domains in ∼2 ps, which is consistent with the reported TA studies of neat Y6 films.^23,31^ The amplitude of the fast PIA decay in the neat film is higher for the 515 nm pump than for the 840 nm pump, suggesting that excitation to a higher energy state or excess vibrational energy assists exciton splitting into charge pairs.

**Figure 6:**
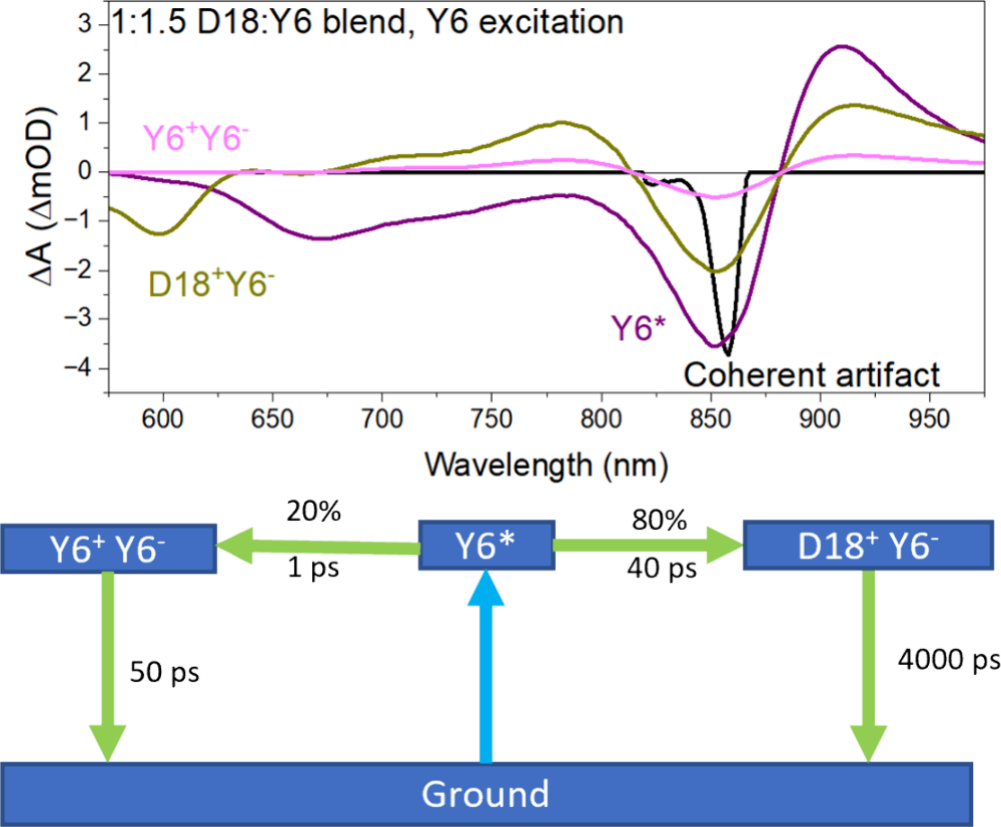
EAS and model of charge generation in 1:1.5 D18:Y6 at 840 nm excitation, with each compartment assigned to various species in the charge generation process.

Other evidence of nonemissive species in neat Y6 films comes from a comparison of the Y6 GSB and PL decays measured at very similar excitation densities (Figure S8). The Y6 GSB shows a fast decay component in ∼50 ps, indicating the presence of nonemissive excited species with a shorter lifetime than excitons. This decay component is independent of excitation density in the range between 2 × 10^17^ and 9 × 10^17^ cm^–3^; therefore, we attribute it to the geminate recombination of charge pairs generated by spontaneous exciton splitting. Enhanced utilization of spontaneously generated charge pairs has been recently reported using n-doping of binary photovoltaic blends with a Y-series acceptor.^26^ Based on these conclusions, we developed a new EAS model to the 1:1.5 blend TA at 840 nm excitation (Figure 6). This model includes parallel de-excitation pathways and assigns each compartment to a species. Initial excitation generates Y6 excitons (Y6*) and a coherent artifact that is removed. Within ∼1 ps, 20% of these excitons spontaneously separate into short-lived charge pairs (Y6^+^ Y6^–^) that recombine over the next 50 ps. Meanwhile, the other 80% of the excitons generate longer-lived charges (D18^+^ Y6^–^) in ∼40 ps, via the previously discussed exciton diffusion and hole transfer mechanism. These long-lived charges generate photocurrent, so we measured the internal quantum efficiency (IQE) of devices for comparison (Figure S1b). The IQE in the region of Y6 absorption is ∼10% lower than in the region of D18 absorption, which is consistent with recombination losses by charge pairs generated in Y6 domains.

Further information about excited states in Y6 domains is obtained by transient anisotropy. The TA kinetics were recorded with the probe polarization set parallel and perpendicular to the pump polarization to give Δ*A*_∥_ and Δ*A*_⊥_, respectively, in a one-color pump–probe experiment. The transient anisotropy *r*(*t*) was then calculated as
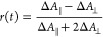
1

**Figure 7:**
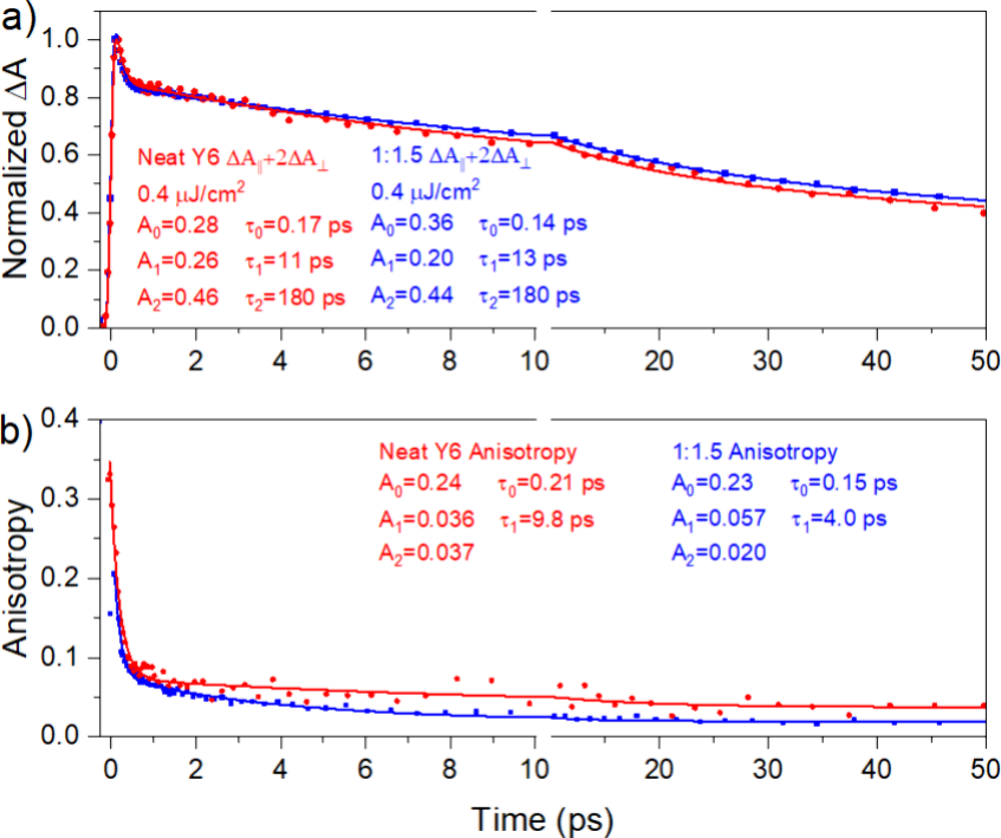
Magic angle kinetics (a) and transient anisotropy (b) in the neat Y6 film and in the 1:1.5 D18:Y6 blend measured in a one-color pump–probe experiment at 860 nm.

For randomly oriented molecules, an initial value *r*(0) = 0.4 is expected if the transition dipole moment experienced by the probe light stays parallel to the transition dipole moment excited by the pump. We observe *r*(0) ≈ 0.3, which decays to *r*(0.2 ps)≈0.1 within our instrument response of ∼200 fs in the blend as well as the neat film (Figure 7). The amplitude of ultrafast depolarization is independent of the pump wavelength in the range 760–860 nm (Figure S9). Therefore, it cannot be explained by incoherent exciton hopping, which would be more pronounced at shorter wavelengths because of the higher spectral overlap of emission from the initially excited molecules with absorption of their neighbors. We attribute this ultrafast depolarization to electronic relaxation and self-trapping of delocalized primary Y6 excitons.^32,33^ Delocalized kinetic Monte Carlo simulations have indicated that a delocalized exciton can split into a charge pair within a single material without energetic offsets,^34^ leading to the ultrafast generation of charge pairs in Y6 domains.

## Discussion

First, we discuss the excitation dynamics following FRET from D18 to Y6. We found that the rise of the D18 hole population closely follows the red shift of the Y6 GSB peak from ∼825 to 847 nm in ∼20 ps (Figure 4b). Kroh et al. showed that the shape of the Y6 ground-state absorption spectrum in the neat film can be fitted to a J-aggregate model, while the dispersed Y6 molecules absorb at much shorter wavelengths, at around 750 nm.^35^ A shoulder at 700–780 nm in the absorption spectrum of the 1:1.5 blend (Figure 1) indicates that some Y6 molecules are less aggregated in the blend as compared to the neat film, which can be explained by the presence of a mixed donor–acceptor phase. Cheng et al. have shown that such a mixed phase coexists with a semicrystalline pure donor phase and a semicrystalline pure acceptor phase in efficient PM6:Y6 blends.^36^ This peak shifts to 820 nm in the first picosecond, indicating fast exciton diffusion to Y6 aggregates. The majority of hole transfer occurs in ∼20 ps after 515 or 640 nm excitation and is mediated by exciton diffusion between Y6 aggregates.

### Driving Force for Hole Transfer

**Figure 8:**
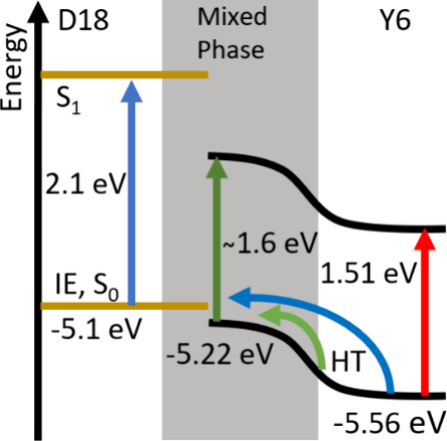
IEs of D18 and Y6 in phase-separated domains and in a mixed phase determined by APS. The blue and green arrows indicate hole transfer pathways from Y6 aggregates to D18 and Y6 in the mixed phase. Optical transitions to the lowest excited state S_1_ in each domain are shown by vertical arrows, with the transition energies taken from the corresponding absorption maxima.

We measured the IEs of neat films and blends by APS (Figure S10). APS, which is also known as photoelectron yield spectroscopy in air or photoelectron spectroscopy in air, measures the current of ionized atmospheric gases by ejected photoelectrons.^27^ It gives us IE values for neat films of 5.56 eV for Y6 and 5.1 eV for D18. These are very similar to the previously reported values using this technique or ultraviolet photoelectron spectroscopy (UPS).^37,38^ The D18:Y6 blend shows the main onset of photoelectron generation at 5.22 eV, which can be attributed to Y6, and a minor onset at 5.1 eV that coincides with that of the neat D18 film. The lower IE of Y6 in the blend as compared to the neat film can be attributed to a mixed phase of Y6 with D18, which forms a “skin” layer at the surface of the blend, similar to what has been observed in other photovoltaic blends.^39−41^ A similar shift of Y6 IE was measured in the PM6:Y6 blend relative to the neat film (∼0.3 eV) by UPS.^42^ The higher IE in the neat Y6 film can be explained by the electrostatic potential formed by the molecular quadrupole moments of aligned Y6 molecules in the semicrystalline phase (Figure 8).^11,12,17^ IE shifts have also been reported in Y6 films deposited from different solvents following changes in aggregation and dominant orientation of Y6 molecules on a substrate.^38^

The driving force for hole transfer is defined as the difference between the Gibbs free energy of the initial and final states, in this case, of the Y6 exciton and the interfacial CT state. The driving force can be estimated using the IE offset between the donor and acceptor and correcting it for the difference of the binding energy of the exciton and the interfacial CT state. In optimized PM6:Y6 blends, the IE offset of ∼0.3 eV was found to be comparable to the binding energy of Y6 excitons, implying a near-zero driving force for hole transfer.^42^ The IE offset of Y6 and D18 in the mixed phase is just 0.12 eV, much smaller than the binding energy of Y6 excitons; therefore, hole transfer in the mixed phase is energetically unfavorable. In contrast, the IE offset of ∼0.46 eV between Y6 aggregates and D18 is larger than the binding energy of Y6 excitons and sufficient to drive hole transfer from Y6 aggregates to D18. According to the Marcus rate equation, the rate of CT scales exponentially with the squared difference between the driving force and the reorganization energy.^43^ Our observation of fast hole transfer in the 1:1.5 blend implies that the driving force is near its optimum for this blend. Similarly, the IE offset of ∼0.34 eV between aggregated Y6 molecules in the semicrystalline phase and dispersed ones in the mixed phase can be sufficient to drive hole transfer and help in the extraction of holes generated by spontaneous exciton dissociation in Y6 domains.

### Blend with a Low Y6 Content

The important role of Y6 aggregates in charge generation is further highlighted by the poor charge generation seen in the 1:0.36 D18:Y6 blend (Figures S3f and S11). In this blend, the ground-state absorption maximum of Y6 occurs at ∼780 nm, indicating that the majority of Y6 molecules are dispersed in the D18 matrix with no significant aggregation. Global analysis of TA spectra in this blend shows the decay of D18 GSB and the rise of Y6 GSB in about 1 ps, which is clear evidence of FRET from D18 to Y6. It is consistent with D18 PL quenching, which also includes a slower decay component of ∼6 ps, with a smaller amplitude representing longer-distance FRET mediated by exciton diffusion (Figure S4a). The Y6 PL is quenched in this blend showing biexponential decay with time constants of 14 and 170 ps and about equal amplitudes (Figure S4b). In contrast, Y6 GSB has a longer lifetime extending into the nanosecond time scale, indicating the formation of nonemissive species (Figure S12). The position of Y6 GSB in EAS **C(1:0.36)** coincides with EAS **A(1:1.5)** of the 1:1.5 blend representing mainly Y6 exciton in the mixed phase after fast FRET from D18 (Figure S11b). However, the **C(1:0.36)** spectrum shows very weak D18 GSB and much less induced absorption compared to **C(1:1.5)** of the 1:1.5 blend, indicating inefficient charge generation in the 1:0.36 blend. The nonemissive species with a nanosecond decay time formed in the 1:0.36 blend could be bound charge pairs generated by electron transfer following D18 excitation. This shows that the ∼0.12 eV IE offset between dispersed Y6 molecules and D18 is not sufficient for carrier generation in blends with D18. This result is consistent with previous findings that a minimum offset of ∼0.3 eV is required for efficient CT at organic heterojunctions.^17,44−48^

### Charge Generation Efficiency

With fully resolved hole transfer dynamics in the 1:1.5 blend, we can estimate the generation efficiency of charges separated into donor and acceptor domains as Φ_gen_ = *k*_HT_/(*k*_HT_ + *k*_eh_), where *k*_HT_ is the rate constant of hole transfer and *k*_eh_ is the decay rate constant of the precursors to separated charges. In this case, precursors include excitons as well as electron–hole pairs formed by spontaneous exciton splitting in Y6 domains. The slowest *k*_HT_ ≈ (30 ps)^−1^ observed with 840 nm excitation in combination with the decay rate of the Y6 exciton from PL measurements *k*_eh_ = (640 ps)^−1^ gives Φ_gen_ ≈ 0.96. The TA measurement of neat Y6 films shows a decay component that can be attributed to the recombination of electron–hole pairs generated in Y6 domains with *k*_eh_ ≈ (200 ps)^−1^ (Figure S8). This rate constant is consistent with a previous report.^49^ If all excitons in the Y6 domains split into charge pairs, then the slow hole transfer measured with 840 nm would strongly limit charge generation to Φ_gen_ ≈ 0.87 of the absorbed near-infrared photons. Shorter excitation wavelengths result in faster hole transfer and higher efficiency; however, harvesting near-infrared photons is particularly important in semitransparent organic solar cells. Improved utilization of spontaneously generated charge pairs has been reported in n-doped binary blends with a Y-series acceptor, which leads to an increased PCE of 18.5%.^26^

### Implications for Devices

The IE offset of 0.46 eV, which drives hole transfer from Y6 aggregates to D18, constitutes the major part of voltage loss, which was estimated at around 0.5 eV in D18:Y6 cells.^13^ Reducing the IE offset by controlling the aggregation of acceptor molecules is possible and may lower the voltage loss, provided it is still sufficient to drive the hole transfer for charge generation. Nonradiative recombination of photogenerated charges through the interfacial CT states also contributes to voltage loss. The energy of these CT states can be estimated by subtracting the IE offset of 0.12 eV in the mixed phase from the optical band gap of Y6 in the mixed phase of ∼1.6 eV, determined from absorption spectra (Figure 8). This brings the CT energy to ∼1.5 eV, very close to that of Y6 excitons in the semicrystalline phase so that a Y6 exciton can be effectively regenerated from the interfacial CT state. The energy proximity of CT and Y6 exciton states is expected to enhance the radiative recombination of CT states, which could then compete with nonradiative recombination, further reducing recombination losses.

Spontaneous exciton splitting into charge pairs in Y6 domains can limit the charge generation efficiency because of the slow hole transfer to D18 and the competing geminate recombination of these charge pairs. Reducing the recombination of charge pairs in Y6 domains or increasing the hole transfer rate is essential to improving device efficiency.

## Conclusions

**Figure 9:**
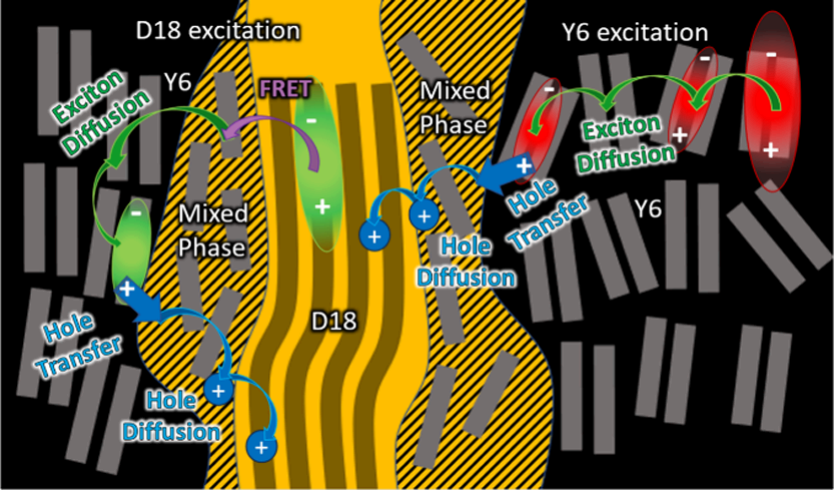
Schematic of the dominant charge photogeneration mechanism in the D18:Y6 blends.

We have found that the dominant charge generation pathway in D18:Y6 solar cells involves energy transfer from D18 to Y6, followed by rapid exciton diffusion to Y6 aggregates in about 1 ps. Slow hole transfer to D18 then occurs on a time scale ranging from a few picoseconds to ∼20 ps (Figure 9). Aggregation of Y6 molecules increases their IE and provides a driving force for hole transfer to D18. The blend with a low Y6 content shows poor charge generation because of the low IE offset of ∼0.12 eV between dispersed Y6 molecules and D18, which is unfavorable for hole transfer. Tuning the IE offset by controlling the aggregation of the low-energy absorber would help balance efficient charge generation with a small voltage loss.

We estimated the energy of interfacial CT states in the mixed D18:Y6 phase to be very similar to that of Y6 excitons in the semicrystalline phase so that Y6 excitons can be effectively regenerated from the interfacial CT states. Energy proximity of CT and Y6 exciton states helps enhance the radiative recombination of CT states and reduce the voltage loss associated with nonradiative recombination.

We observed ultrafast depolarization of the Y6 GSB in <200 fs, which indicates delocalization of primary excitons in Y6. This delocalization can explain the ultrafast generation of charge pairs in neat Y6 films and Y6-rich blend domains, which enables exciton harvesting in semitransparent solar cells with a low donor content. It is important to increase the hole diffusion length in Y6 to prevent the geminate recombination losses in these blends. This charge generation mechanism is advantageous for reducing voltage loss to the nonradiative recombination of interfacial charge pairs. Relatively slow hole transfer to D18 leads to losses associated with the geminate recombination of charge pairs generated in Y6 domains. Reducing the recombination of the charge pairs in Y6 domains is important for improving device efficiency.

## Supporting Information Available

Device characteristics of D18:Y6 solar cells; absorption, transient absorption, and time-resolved photoluminescence of various blends and neat films of D18 and Y6; and derivation of diffusion length and average distance between the donor and acceptor. Data supporting this publication is available from the University of St-Andrews data repository at DOI: 10.17630/b95ac71a-a36b-47c2-8799-b943774f8d45 (PDF)
