# Supplementary material for: Exciton Diffusion to Low Energy Sites of the Acceptor Drives Charge Photogeneration in D18:Y6 Solar Cells
Source: J Phys Chem C Nanomater Interfaces. 2024 Oct 31;128(45):19319–28. doi: 10.1021/acs.jpcc.4c06706 (PMC11571220; doi:10.1021/acs.jpcc.4c06706)
Supplement: Supplementary file 1 — jp4c06706_si_001.pdf [file jp4c06706_si_001.pdf]

Exciton Diffusion to Low Energy Sites of the Acceptor Drives Charge Photogeneration in D18:Y6 Solar Cells

Supplementary Information

Thomas Sayner, Arvydas Ruseckas, Jonathon R. Harwell, Ifor D. W. Samuel

Organic Semiconductor Centre, SUPA, School of Physics and Astronomy, University of St. Andrews, North Haugh, St. Andrews, Fife KY16 9SS, United Kingdom

Table S1. Characteristics of D18:Y6 solar cells with blend ratio of 1:1.8 prepared with different spin speeds, SVA means solvent vapor annealing in chloroform for 5 minutes. The average parameters and standard deviations were calculated from eight cells.

Spin speed, annealing	PCE maximum (%)	PCE average (%)	Fill Factor average (%)	Short circuit current average (mA/cm^2)	Open circuit voltage average (V)
4000 rpm, with SVA	15.9	15.0 ± 0.7	73.0 ± 1.0	-24.5 ± 1.2	0.837 ± 0.003
4000 rpm, no SVA	15.9	14.7 ± 0.9	73.1 ± 0.5	-23.8 ± 1.4	0.841 ± 0.004
1500 rpm, with SVA	14.2	13.6 ± 0.4	67.3 ± 1.1	-24.5 ± 0.4	0.822 ± 0.003
1500 rpm, no SVA	13.9	13.3 ± 0.3	67.5 ± 1.2	-24.0 ± 0.7	0.819 ± 0.003

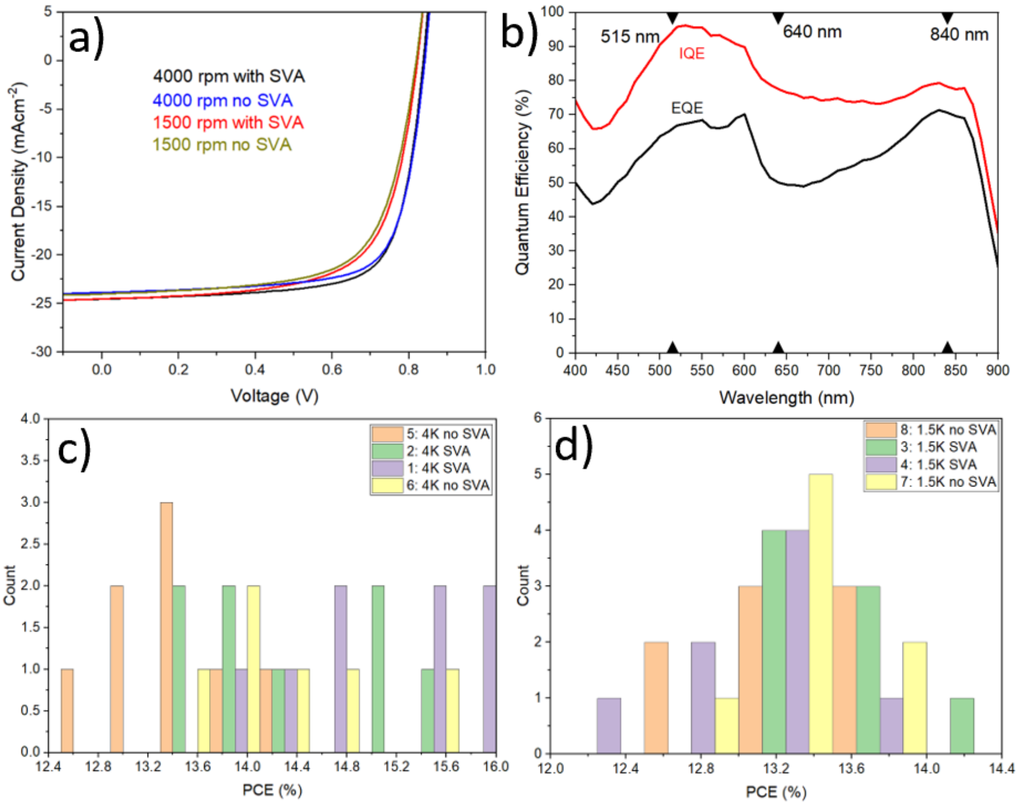

Figure S1. (a) J-V curves of D18:Y6 1:1.8 solar cells under AM1.5G illumination (100 mW/cm^2) averaged over eight pixels with pixel area of 0.05 cm^2. Active layers were spin-coated with the spin speeds indicated, SVA is solvent vapor annealing in chloroform for 5 minutes. (b) EQE and IQE of devices. Histogram of PCE counts for devices spin coated at (c) 4000 rpm and at (d) 1500 rpm.

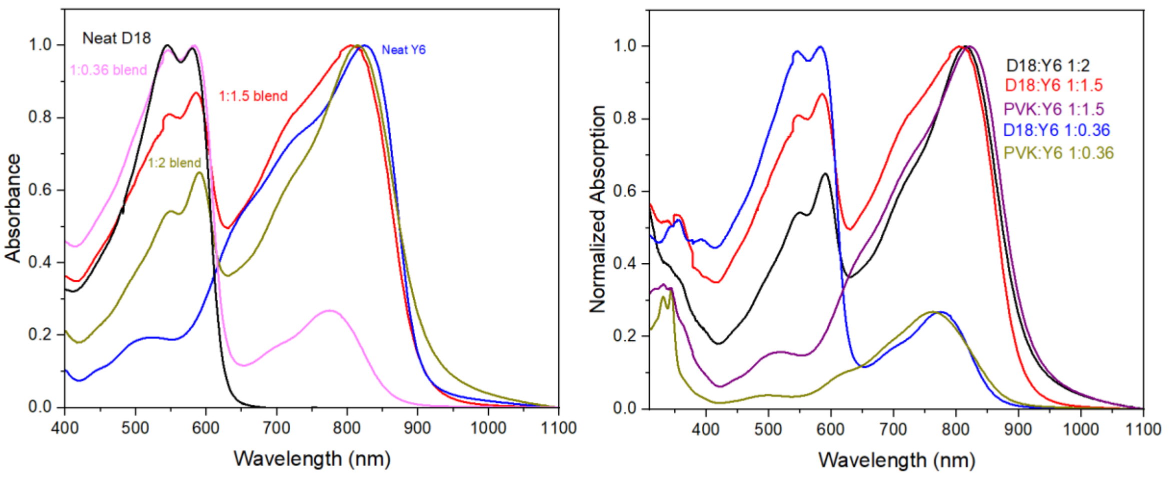

Figure S2. Absorption spectra of D18:Y6 (left) and PVK:Y6 (right) blends with different blend ratios and of neat films.

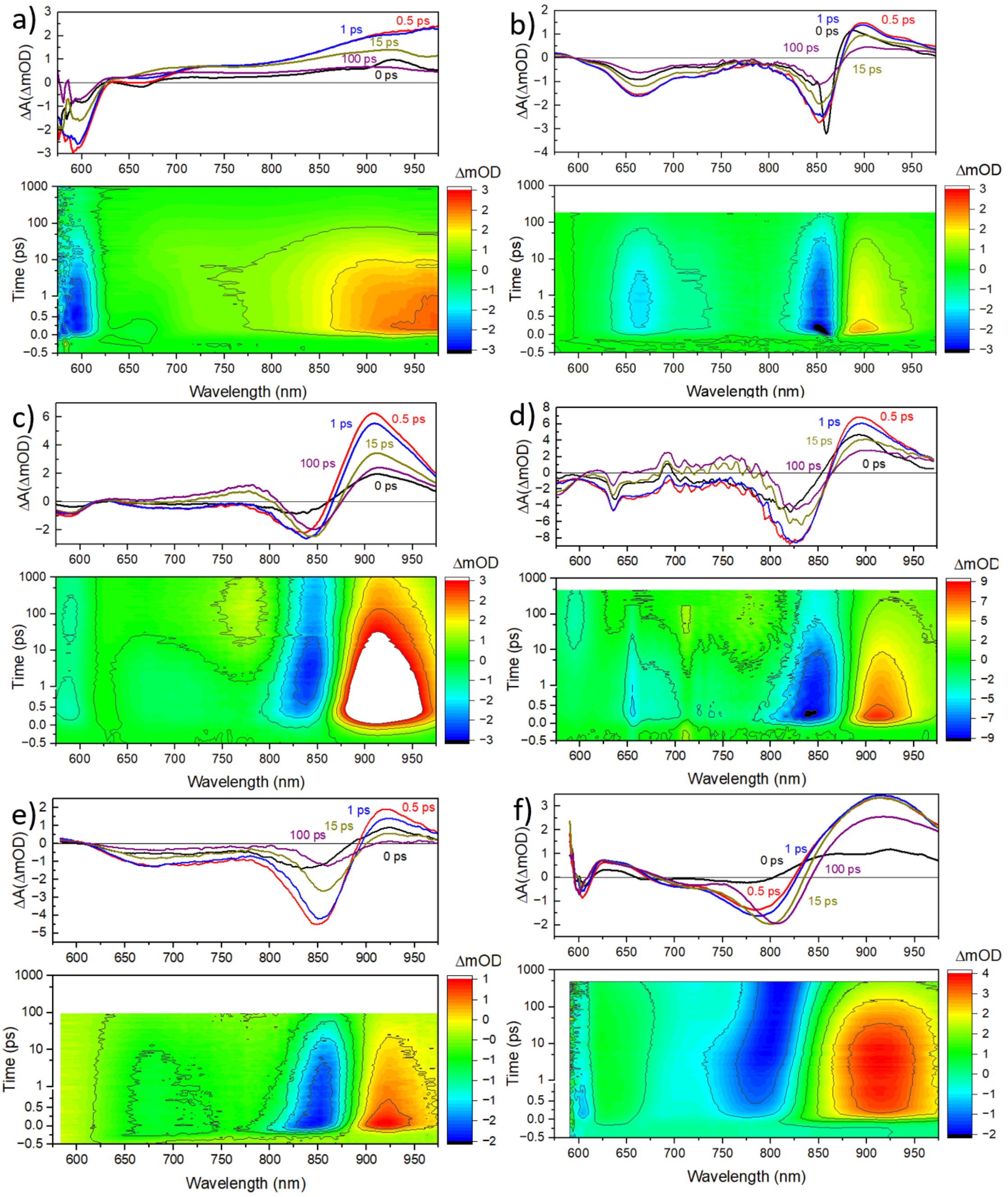

Figure S3. Transient absorption spectra and carpet at 515 nm excitation for (a) neat D18 4.8 μJ/cm^2, (b) neat Y6 (840 nm excitation) 2 μJ/cm^2, (c) 1:2 D18:Y6 blend 2 μJ/cm^2, (d) 1:1.5 blend (640 nm excitation) 3.4 μJ/cm^2, (e) 1:1.5 PVK:Y6 blend 3 μJ/cm^2, (f) 1:0.36 D18:Y6 blend 4.8 μJ/cm^2.

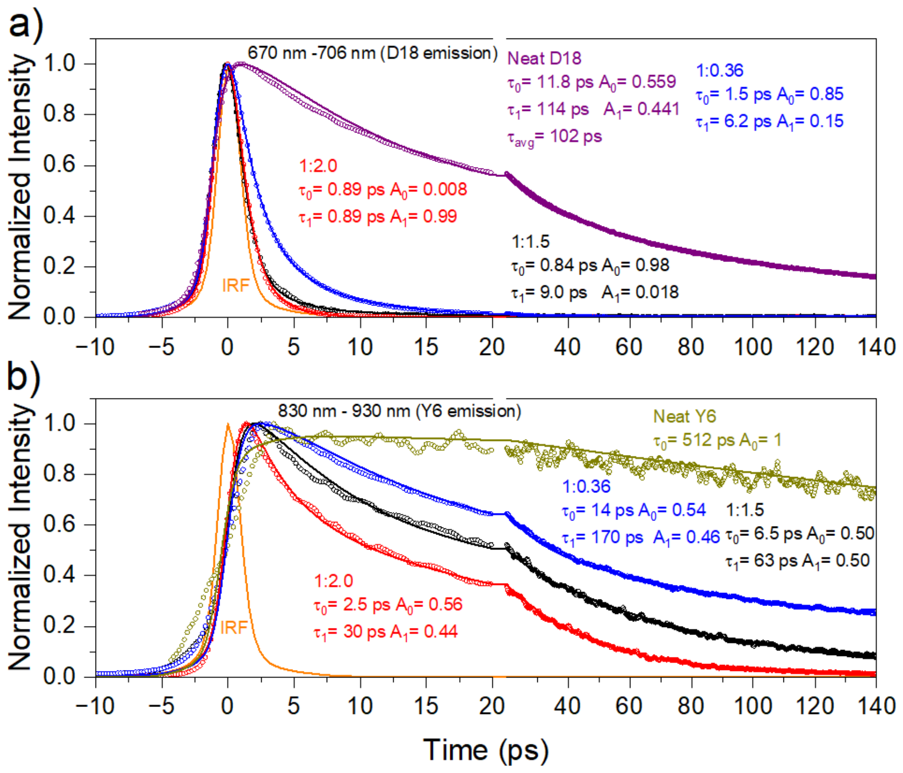

Figure S4. Photoluminescence decays in neat films and D18:Y6 blend films of different ratios detected in the range of 670 nm – 706 nm (D18 emission, a) and 830 nm – 930 nm (Y6 emission, b) with excitation at 515 nm. IRF is the instrument response function. Insets show the time constants and amplitudes of biexponential decay fits which are presented as solid lines.

Table S2: Goodness of fit of the transient absorption with evolution-associated spectra (EAS).

Fit	χ^2
D18:Y6 1:1.5 515 nm excitation	$1.15x10^{-2}$
D18:Y6 1:1.5 840 nm excitation	$2.73x10^{-3}$
D18:Y6 1:0.36 515 nm excitation	$1.16x10^{-2}$
Neat D18 515 nm excitation	$1.61x10^{-2}$
Neat Y6 515 nm excitation	$1.12x10^{-1}$

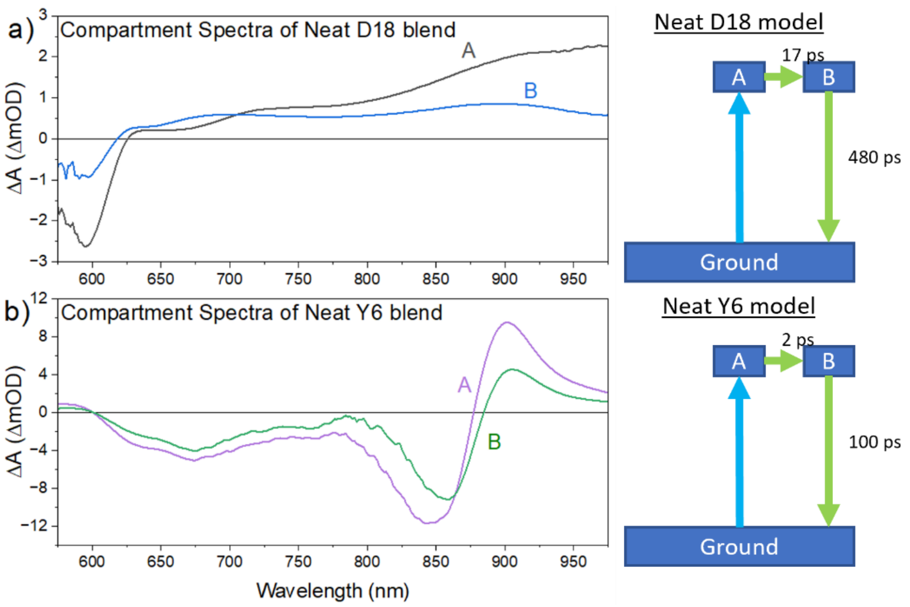

Figure S5. Evolution-associated spectra and model capturing excited-state dynamics in (a) neat D18 and (b) neat Y6 films under 515 nm excitation.

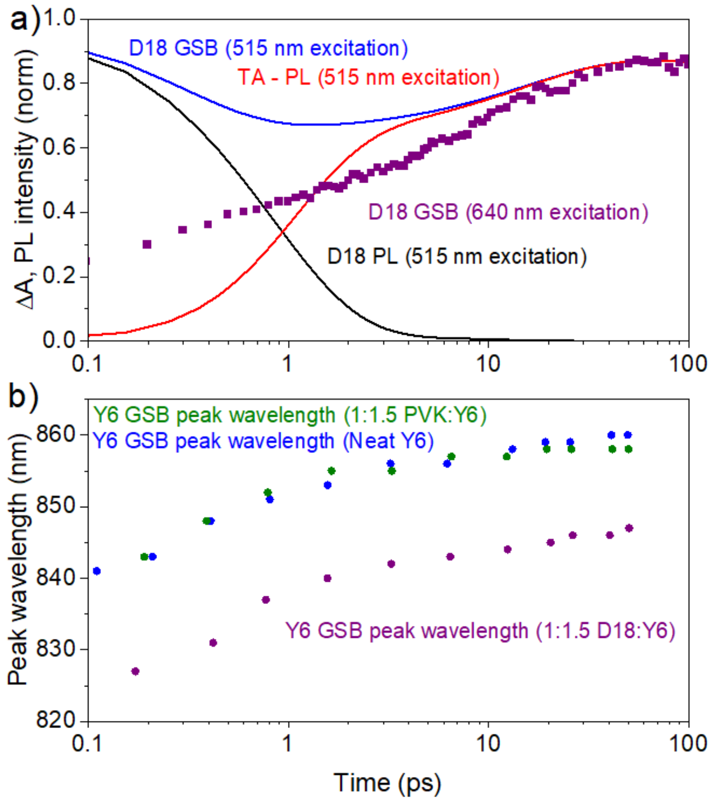

Figure S6. a) Deconvoluted kinetics of D18 GSB at 570 nm and photoluminescence decay of D18 in the 1:1.5 blend with 515 nm excitation (lines). TA-PL is the difference between them which represents the hole population in D18 after 515 nm excitation. Symbols show D18 GSB after 640 nm excitations. (b) Peak shift of Y6 GSB in neat Y6 film and in the blends with D18 and PVK.

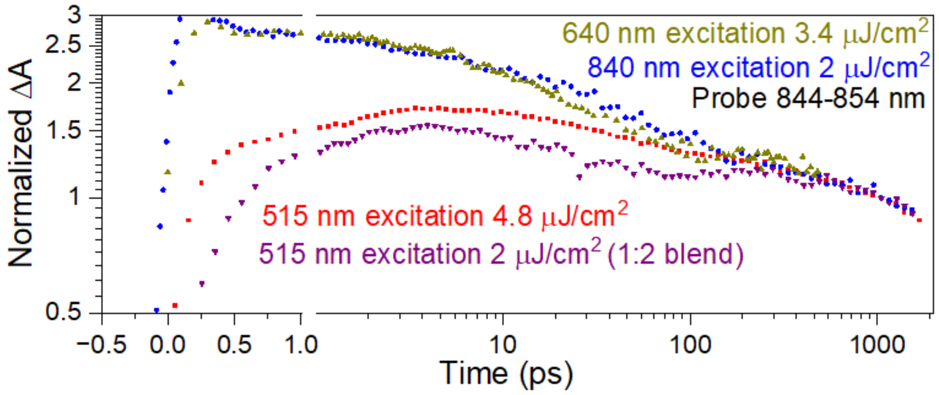

Figure S7. Transient evolution of the Y6 GSB (844-854 nm) of the 1:1.5 blend at 515 nm, 640 nm and 840 nm excitation. Also shown is the 1:2 blend at 515 nm excitation.

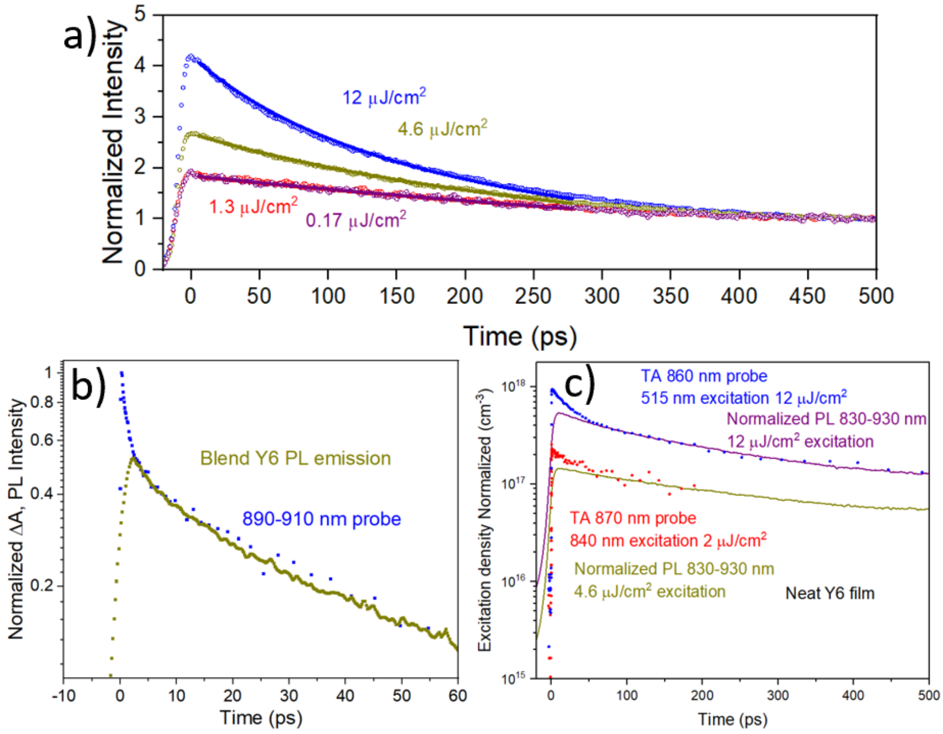

Figure S8. (a) PL decays of neat Y6 at different excitation densities. (b) TA kinetics at 890-910 nm in the 1:1.5 blend with the long-lived component representing absorption by photogenerated charges subtracted and photoluminescence decay of Y6 for 515 nm excitation. The ultrafast decay component of PL in < 2ps is not resolved because of limiting instrument response. Beyond 2 ps both decays are similar indicating that the photoinduced absorption at 890-910 nm up to 60 ps is due to Y6 excitons. (c) Kinetics of Y6 GSB (symbols) and PL decays in neat Y6 film measured at very similar excitation densities normalized at times after 100 ps (lines).

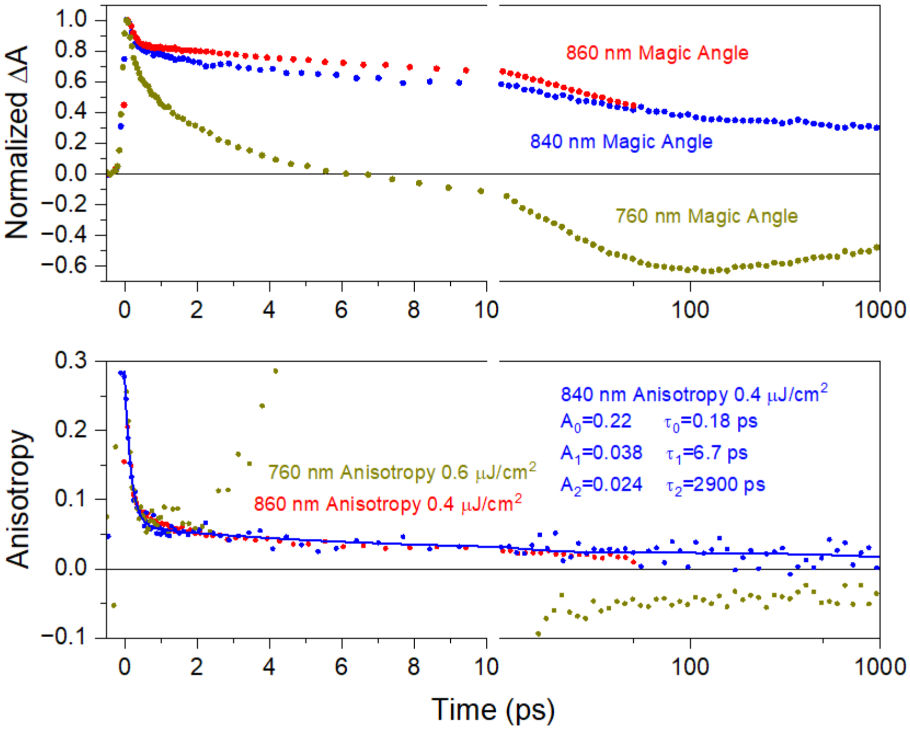

Figure S9. One-color pump-probe kinetics at the magic angle (top) and transient anisotropy (bottom) in 1:1.5 blend for different wavelengths.

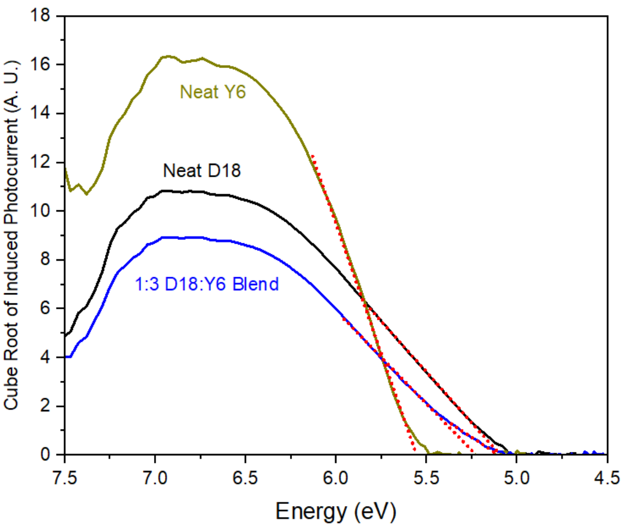

Figure S10. Air photoemission spectra of neat films and the D18:Y6 blend. The ionization energies were determined from the slope intersects shown by dotted lines.

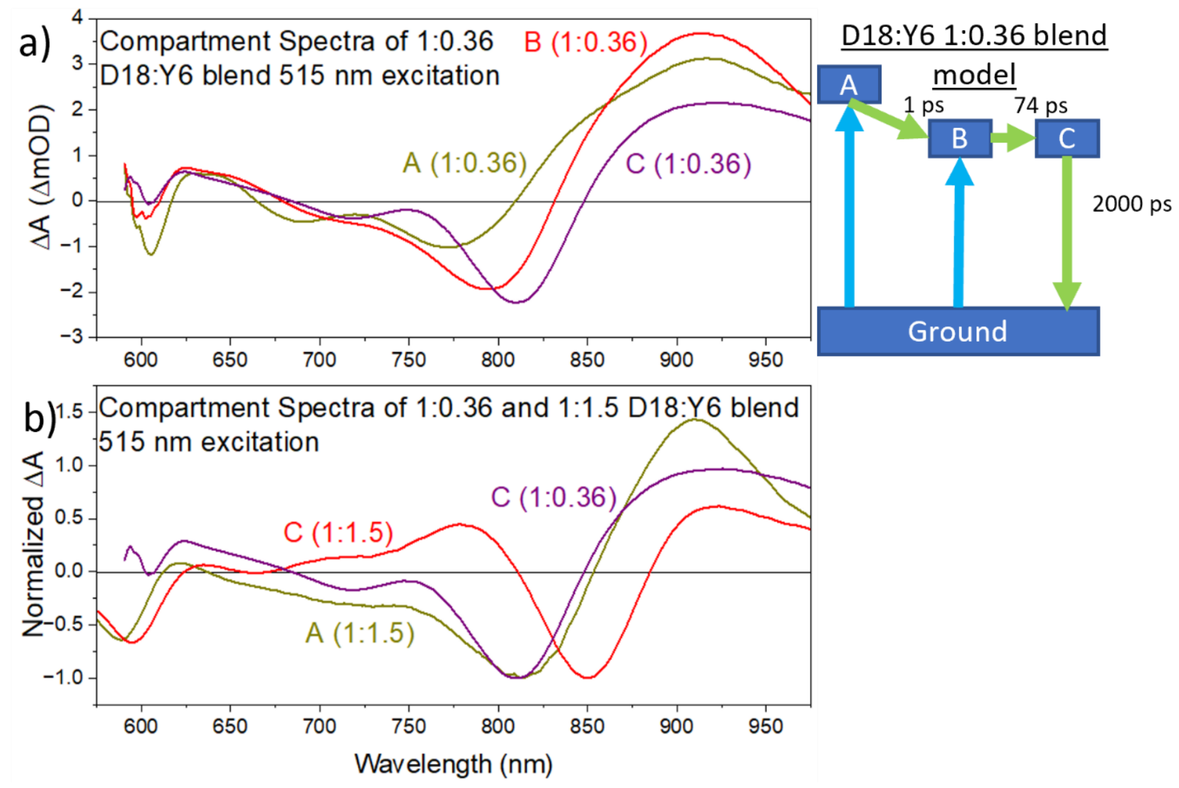

Figure S11. (a) EAS and model of the blend with low Y6 content. (b) EAS of the long-lived excitation in the blend with low Y6 content C(1:0.36) compared to A(1:1.5) which is dominated by Y6 exciton in the mixed phase after FRET from D18 and to C(1:1.5) which represents generated hole polaron in D18 and electron polaron in Y6. This shows that non-aggregated Y6 molecules do not support hole transfer to D18.

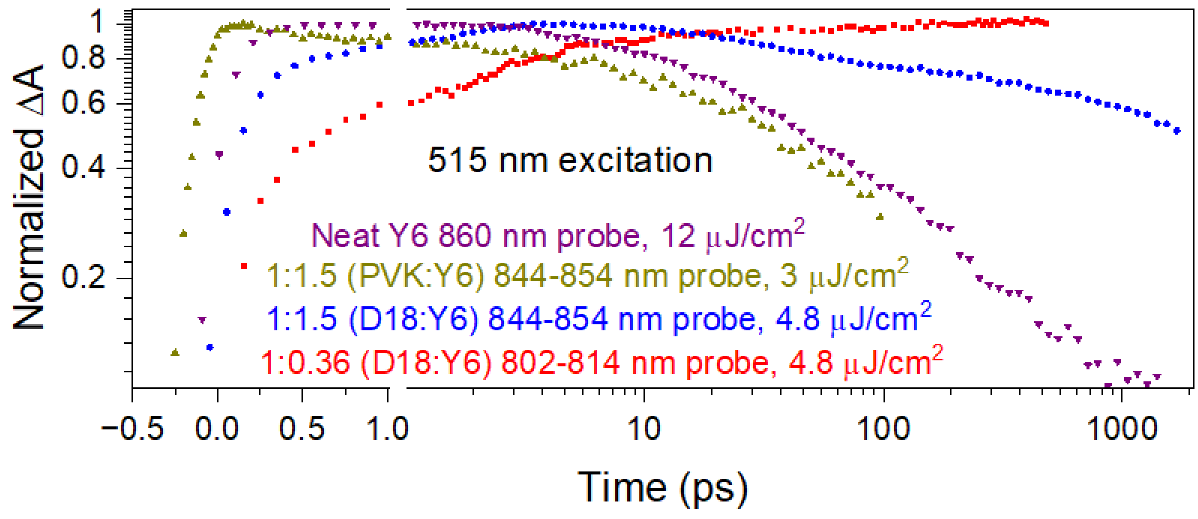

Figure S12. Transient evolution of the Y6 GSB at 515 nm of neat Y6, 1:0.36 D18:Y6, and 1:1.5 blends of D18:Y6 and PVK:Y6 at 515 nm excitation.

Supplementary note 1. Estimation of the D18 domain width

We estimated the average size of D18 domains using the observed FRET rate. To account for the size of Y6 domains we used a FRET model from a point dipole to a slab. The critical transfer distance (analogous to the Förster radius) from a donor molecule to an infinitely thick layer of acceptor d_∞ can be described by[1]

$$d_\infty^3 = \frac{3\eta}{2(4\pi n)^4} \int A(\lambda) f(\lambda) \lambda^4 \, d\lambda \qquad (1)$$

where η is the photoluminescence quantum yield (PLQY) of the donor, which we measured to be 0.131 for neat D18 film. A and n are the absorption per unit length and refractive index of the film respectively. This was obtained from work by Kerremans et al, with n≈2 used for the spectral overlap range 570-850 nm.[2] The range was determined from the regions where the Y6 absorption A(λ) and D18 photoluminescence emission f(λ) overlap (Fig. S13).

The critical transfer distance d_0 to a finite layer of acceptor with the thickness Δ can be determined from

$$\frac{1}{d_\infty^3} = \frac{1}{d_0^3} - \frac{1}{(d_0+\Delta)^3} \qquad (2)$$

Here we used the π-π stacking coherence length of Y6, Δ ≈ 2.7 nm, reported by Wang et al[3] to determine d_0=8.5 nm.

The distance z between donor and acceptor can then be found by solving:

$$k_{FRET}(z) = \frac{d_0^3}{\tau}\left[\frac{1}{z^3} - \frac{1}{(z+\Delta)^3}\right] \qquad (3)$$

Here τ=102 ps is the average PL lifetime of neat D18. We use the average time constant of PL decay in the blend for $k_{FRET} \approx 1\times10^{12}$ s^{-1} This gives an average FRET distance z ≈1.8 nm and pure D18 domain width 2z≈3.6 nm. This is identical to the π-π stacking coherence length of

D18 chains found in D18:Y6 blend by Wang et al using GIWAXS measurements.[3] This agreement indicates similar blend morphology of our blends and the published work. Domain width of 3.6 nm corresponds to about nine stacked D18 chains indicating phase separation on an intermediate scale in this blend.

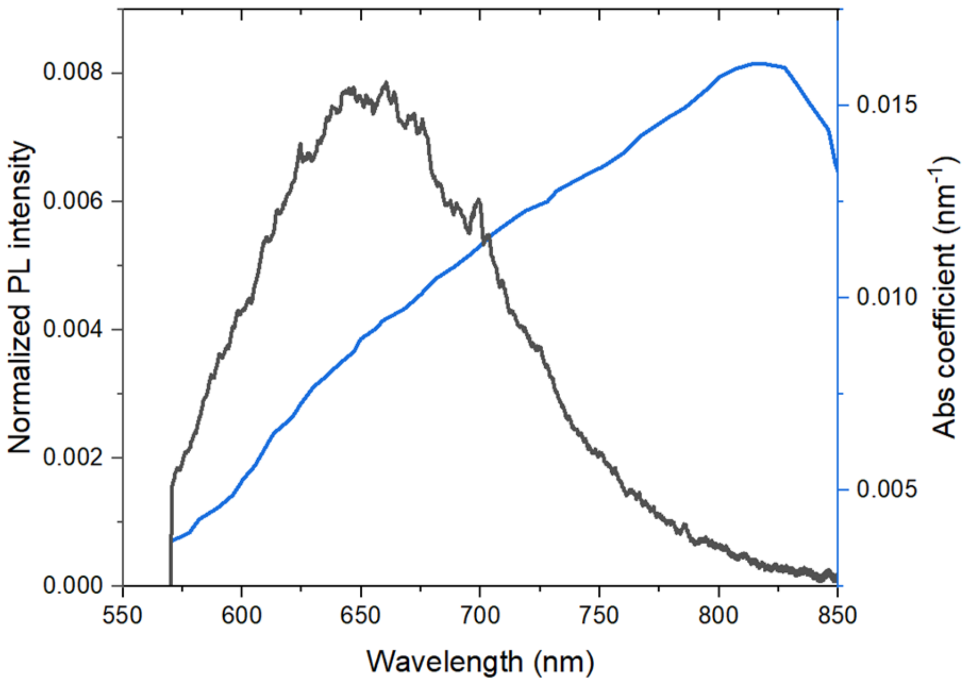

Figure S13. Absorption coefficient of Y6 film and PL spectrum of D18 films.

Supplementary note 2. Estimation of IQE.

IQE was determined by dividing EQE by the fraction of absorbed light. This fraction was estimated by simulating the solar cell stack using MATLAB code made available by Burkhard et al, using optical constants measured via spectroscopic ellipsometry.[4]

References

(1) Shaw, P. E.; Ruseckas, A.; Samuel, I. D. W. Distance Dependence of Excitation Energy Transfer between Spacer-Separated Conjugated Polymer Films. *Phys. Rev. B* **2008**, *78* (24), 245201. https://doi.org/10.1103/PhysRevB.78.245201.

(2) Kerremans, R.; Kaiser, C.; Li, W.; Zarrabi, N.; Meredith, P.; Armin, A. The Optical Constants of Solution-Processed Semiconductors—New Challenges with Perovskites and

Non-Fullerene Acceptors. *Adv. Opt. Mater.* **2020**, *8* (16), 2000319. https://doi.org/10.1002/adom.202000319.

(3) Wang, Z.; Peng, Z.; Xiao, Z.; Seyitliyev, D.; Gundogdu, K.; Ding, L.; Ade, H. Thermodynamic Properties and Molecular Packing Explain Performance and Processing Procedures of Three D18:NFA Organic Solar Cells. *Adv. Mater.* **2020**, *32* (49), 2005386. https://doi.org/10.1002/adma.202005386.

(4) Burkhard, G. F.; Hoke, E. T.; McGehee, M. D. Accounting for Interference, Scattering, and Electrode Absorption to Make Accurate Internal Quantum Efficiency Measurements in Organic and Other Thin Solar Cells. *Adv. Mater.* **2010**, *22* (30), 3293–3297. https://doi.org/10.1002/adma.201000883.
